# An Open Source Syringe Pump Controller for Fluid Delivery of Multiple Volumes

**DOI:** 10.1523/ENEURO.0240-19.2019

**Published:** 2019-09-06

**Authors:** Linda M. Amarante, Jonathan Newport, Meagan Mitchell, Joshua Wilson, Mark Laubach

**Affiliations:** 1 Center for Behavioral Neuroscience, American University, Washington, DC 20016; 2 Department of Physics, American University, Washington, DC 20016

**Keywords:** fluid, licking, open-source, reward, sucrose, value

## Abstract

Syringe pumps are a necessary piece of laboratory equipment that are used for fluid delivery in behavioral neuroscience laboratories. Many experiments provide rodents and primates with fluid rewards such as juice, water, or liquid sucrose. Current commercialized syringe pumps are not customizable and do not have the ability to deliver multiple volumes of fluid based on different inputs to the pump. Additionally, many syringe pumps are expensive and cannot be used in experiments with paired neurophysiological recordings due to electrical noise. We developed an open source syringe pump controller using commonly available parts. The controller adjusts the acceleration and speed of the motor to deliver three different volumes of fluid reward within one common time epoch. This syringe pump controller is cost effective and has been successfully implemented in rodent behavioral experiments with paired neurophysiological recordings in the rat frontal cortex while rats lick for different volumes of liquid sucrose rewards. Our syringe pump controller will enable new experiments to address the potential confound of temporal information in studies of reward signaling by fluid magnitude.

## Significance Statement

The neural basis of reward processing is a major topic in neuroscience. Many studies examine reward coding by varying the amount of fluid given to experimental animals as they perform a behavioral task. Most devices used in these studies are not capable of delivering different volumes over a common period of time. As such, reward coding is confounded by temporal information. To resolve this issue, we developed a syringe pump controller that is capable of delivering different fluid volumes, in the range used in behavioral studies, over common periods of time. Here, we report the designs of all components of the pump and the protocols needed to create and use it.

## Introduction

In behavioral neuroscience research there is often a need to deliver fluid rewards to animals. This is typically done using passive gravity-based systems, syringe pumps, or solenoid valves. Syringe pump systems can be quite costly when purchased from a commercial vendor (>$500) and are often limited in their functionality. Commercial laboratory equipment is also not currently available to deliver multiple fluid volumes on a microliter scale. Instead, different fluid volumes are produced by using syringes of different sizes from different pumps or by leaving a syringe pump running or solenoid valve open for different periods of time. As such, published neuroscience studies of reward magnitude coding have confounded fluid volume with reward delivery time (Fiorillo et al., 2003; Bermudez and Schultz, 2010) through using longer solenoid valve openings to give rewards of different sizes to animals. Therefore, previous findings on neurophysiological correlates of reward magnitude encoding may potentially be due to the timing of reward delivery, not the size of the reward. This highlights the need for a syringe pump that can adequately deliver multiple fluid volumes over a common time epoch.

To address this issue, we developed an open source syringe pump control circuit using commonly available parts and a Teensy microcontroller with open source firmware. We modified an open source 3D printed syringe pump holder (Wijnen et al., 2014) to house the syringe (though any syringe pump holder will do) and built a new circuit to control the motor of the syringe pump. We created a printed circuit board (PCB) that can take multiple inputs to deliver up to three different fluid volumes from one syringe over a common time epoch. The functionality of the pump is achieved by changing the velocity of the motor and acceleration through a custom algorithm within the firmware. The syringe pump controller is time precise in that it delivers a bolus of fluid across the user-specified time epoch. Notably, the circuit board does not produce excess electrical noise, making it an optimal tool to pair with electrophysiological recordings.

Here, we provide instructions for building the syringe pump controller and share the printed circuit board design, parts list, and microcontroller firmware for developing the syringe pump controller, all of which can be made for <$300. We validated the syringe pump controller by implementing it in a sucrose licking task where rats licked for different volumes of fluids, and show that neural activity in the medial frontal cortex can simultaneously be recorded while using the syringe pump controller to deliver rewards of different sizes.

## Materials and Methods

### Syringe holder

To hold the syringe, we used a published 3D printed syringe pump design (Wijnen et al., 2014), and included modifications made by the original authors. However, any syringe holder that can fit a 30–60 ml syringe will work with the syringe pump controller. We made additional modifications to the design by Wijnen et al. (2014) to accommodate a NEMA-17 integrated stepper motor+driver, and for the pump holder to fit both a 30 and a 60 ml syringe. We also adjusted the end pieces to have a more stable base for the holder (see Fig. 3). The .stl files of the 3D printed parts for a 60 ml syringe holder can be found on GitHub https://github.com/LaubachLab.

### Bill of materials

A bill of materials for constructing the syringe pump controller can be found as a spreadsheet in Extended Data 1 and on GitHub https://github.com/LaubachLab.

10.1523/ENEURO.0240-19.2019.ed1 Extended Data 1 Teensy Code Firmware (.ino); PCB design files (.brd, .sch, .pdf) and libraries in Eagle (.lbr); Syringe calibration spreadsheet for water and 16% sucrose (.xlsx); Build instructions (.pdf); Bill of materials (.xlsx); Closeup images of the device (.png). Download Extended Data 1, ZIP file.

### Syringe pump control board build and programming instructions

**Figure 1.**
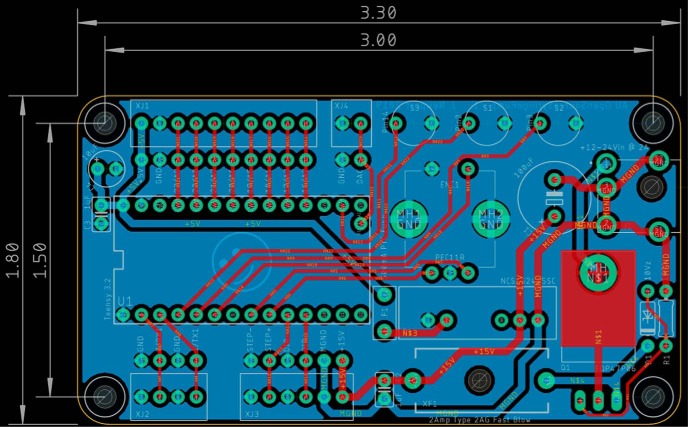
PCB. Dimensions and schematic of PCB for the syringe pump controller.

The schematic for the PCB (Fig. 1) and manufacturing files are available in Extended Data 1 and on GitHub https://github.com/LaubachLab. PCBs were designed using AutoCAD Eagle software (Autodesk). Boards are also available directly from various PCB manufacturing houses, including for purchase on the project page of OSH Park https://oshpark.com/shared_projects/VtseE2Ia.

**Figure 2.**
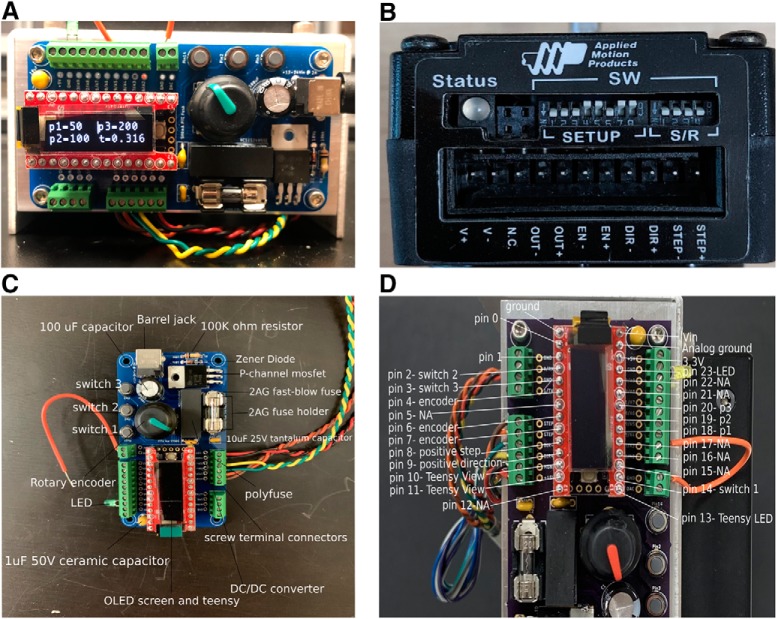
The syringe pump controller and motor. ***A***, The syringe pump controller. When plugged into a power supply, the OLED screen displays four variables: p1, p2, p3 (indicating the number of steps issued to the motor), and *t* (fluid delivery time). These can be changed through using the rotary encoder to the right of the OLED screen. ***B***, The motor jumpers are set so that for every 200 pulses issued to the motor, the motor shaft will rotate 360°. Each single pulse or step will rotate 360°/200 = 1.8°. ***C***, Pinout of the controller with the location of various parts on the PCB. ***D***, Pinout details for the teensy microcontroller.

**Figure 3.**
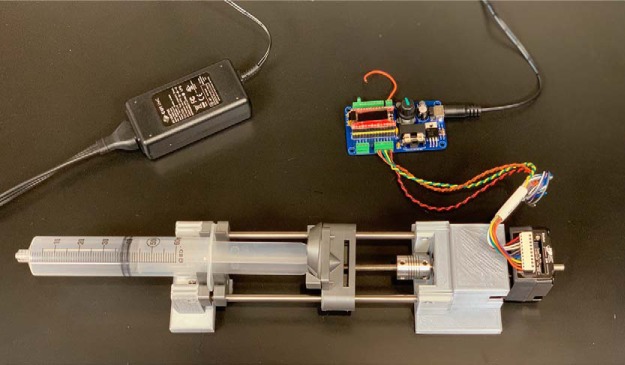
Device setup. View of a 60 ml syringe pump inside a 3D printed holder. This is attached to the NEMA 17 integrated motor+driver, which is attached to the syringe pump controller, powered off of a 15 V power supply.

A detailed protocol for constructing the syringe pump controller can be found in Extended Data 1 and on GitHub https://github.com/LaubachLab. An example of the control board is shown in Figure 2, and the entire syringe pump setup is shown in Figure 3.

### Velocity profile algorithm

The syringe pump controller delivers dissimilar volumes of fluid in equal time intervals. To achieve this, we used formulae derived from the kinematics of the stepper motor driving the linear motion of the syringe plunger.

The angle of the output shaft of the motor is controlled by digital pulses issued by a microcontroller. A typical stepper motor is designed with 200 steps/revolution (or 1.8°/step). The linear motion of the syringe plunger is directly proportional to the angle of the output shaft of the motor. Consequently, the amount of fluid delivered is a function of this angle and the size of the syringe. As such, the kinematic equations are derived using *x* (number of steps) as the position variable, *v* (steps per second) as the velocity variable, and *a* (steps per second squared) as the acceleration variable. The amount of fluid delivered per step must be determined via a calibration procedure for a particular syringe design as detailed below.

When delivering a specified volume of fluid, the stepper motor driving the linear motion of the syringe plunger undergoes three stages of motion—initial acceleration, constant velocity, and final deceleration. Given a fixed acceleration and the maximum desired number of steps, *x*_max_, the minimum amount of time required to accelerate to a maximum velocity and then immediately decelerate to zero velocity is given by:

$$t_{min}=\sqrt{\frac{4x_{max}}{a}}.$$

Given the same acceleration and fixed *t*_min_, we may now calculate the velocity *v_n_* for the desired number of steps, *x_n_*, less than *x*_max_, as follows:

$$v_{n}=\frac{at_{min}-\sqrt{a^{2}t_{min}^{2}-4ax_{n}}}{2}.$$

Therefore, given a fixed acceleration and variable distances (less than some maximum distance traveled), velocity profiles can be generated that ensure desired fluid volume delivery over an equal time interval.

### Basic instructions for operation

Perform the steps in the Calibration methods section to obtain fluid volumes based on the user’s individual setup. (This is a necessary step, considering that the size, diameter, and material of the syringe as well as the type of motor or syringe holder could alter the exact fluid volume dispensed).

Power up the device using a 12–24 volt DC power supply.

Check that the values that appear on the organic light-emitting diode (OLED) screen are correct for the desired volume dispensed, according to syringe pump calibration.

Load up the syringe with fluid. The motor can be advanced in either direction by pressing the switch buttons on the board so that the pusher block is flush with the plunger in the syringe.

Any input to the pins on the board (pin 18, 19, or 20) from behavioral software or from pressing switch buttons will dispense fluid.

The volume of fluid delivered by the syringe pump depends on the size of the syringe, the pitch of the pump leadscrew, and the total rotation of the motor. Stepper motor rotation is defined by the number of “steps” issued to the motor controller. If the jumper switches are set as shown in Figure 2*B*, for every 200 pulses issued to the motor via the “STEP+” and “STEP−” pins the motor shaft will rotate 360°. If a single pulse or step is issued, the shaft will rotate 360°/200 = 1.8°. The OLED screen displays three values (p1, p2, and p3) indicating the number of steps that can be issued to the integrated motor over an equal time period (*t*).

### How to change the reward volume

The three motor rotation values are activated by a falling voltage transition on pins 18, 19, and 20, which then rotates the motor shaft p1, p2, and p3 steps, respectively. Additionally, these step sequences can be activated simply by connecting pins 18, 19, and 20 to the GND (ground) pin on the circuit board. For example, if p2 = 100 and pin 19 is connected to the GND pin, the motor will rotate 100 steps, which for the default setup is 180°. Given that a different size or different diameter syringe will dispense different fluid, it is necessary to calibrate the syringe controller with the desired type of syringe (see Calibration methods).

After connecting the controller to a power supply, there are four variables that appear on the OLED screen: p1, p2, p3, and t. p1, p2, and p3 equal different “steps” or rotations that the motor will advance by each time it receives a falling edge input to pins 18, 19, or 20 on the circuit board. By default, these are set in the Teensy code to p1 = 50, p2 = 100, and p3 = 200. These values can be changed either within the code or by using the rotary encoder. Pressing down on the rotary encoder will change between the values and rotating the knob will alter the selected value. The settings will revert to the default numbers every time the pump is unplugged. The actual volume of fluid dispensed will depend on the type of syringe used and the viscosity of the fluid, which can be determined through performing pump calibration measurements as shown in Extended Data 1.

### How to change the reward timing

The timing of the reward (variable *t* on the TeensyView OLED screen) is determined by the fixed acceleration of the motor and the third variable, p3. The value of the variable p3 will designate the timing of the reward (*t*), and then the acceleration for variables p1 and p2 is automatically adjusted so that p1 and p2 will deliver their respective volumes along the same time scale as p3. These variables can all be changed either within firmware or on the rotary encoder, as seen in Figure 2*A*. In Figure 2*A*, setting p3 to 200 will deliver the largest volume across 316 ms. The smallest volume reward is determined by p1, and will deliver 25% of the volume as p3. Setting p2 will deliver an intermediate volume reward that is half the size of the corresponding volume for p3, and all volume rewards will be delivered across 316 ms. As the minimum time is set by the maximum distance traveled, p1, p2, and p3 can be changed to their respective numbers representing the desired volumes (Extended Data 1, calibration spreadsheet). For example, if the integrated motor set switches are set to 200 steps/revolution, the acceleration is set to stepAccel = 8000 steps/s^2^ and p3 = 200 steps, and the motor will rotate once in 316 ms.

The timing is highly dependent on the motor control algorithms. For example, the standard Arduino stepper motor control libraries (e.g., Stepper) yielded timing up to 15% lower than the theoretically calculated values. Accurate timing information is available via the serial port or pin 23 on the controller board.

### How to connect to behavioral equipment

The device can take several inputs from behavioral software. As stated above, the inputs read a falling voltage transition. The circuit board and firmware are programmed to take three inputs that each wait for a falling edge transition to be activated (pins 18, 19, and 20). Conversely, pin 23 is programmed as an output to go HIGH (3.3 V on a Teensy 3.2 microcontroller) when the motor is moving and LOW (0 V) when the motor stops. An LED may be connected to this pin to indicate motor movement or it may be used to pass accurate timing information to other instruments.

Several additional general purpose input/output analog inputs and the onboard digital-to-analog converter of the microcontroller are available for controlling additional hardware or accepting inputs from switches or sensors. The firmware is easily modified and programmed using the Arduino Software IDE.

### Additional benefits

There are additional advantages that come with using a NEMA-17 integrated stepper motor+driver with this control circuit. Previous syringe pump designs have made it difficult to drive the pump in reverse or pull back the plunger of the pump to use for suction instead of fluid delivery. The integrated motor has functionality to run forward or backyard through pressing one of three push-button switches on the board. Holding down one of the three push-button switches (pin14, pin2, and pin3) will move the pump in a clockwise direction or a counterclockwise direction, or will cycle the pump through a short dance of the programmed values, respectively. The integrated stepper motor driver is optimal to use with the controller instead of a standard NEMA 17 motor since the integrated motor driver will provide accurate motor rotation and will therefore provide reliable fluid volumes.

### Validation

The syringe pump control circuit was tested first to validate adequate changes in fluid volumes at each step. We created a spreadsheet of the exact volumes delivered with 30 and 60 ml syringes, first with water and then additionally with 16% sucrose, which is commonly used in our behavioral experiments. We then validated the use of the pump in a behavioral experiment paired with electrophysiological recordings in a group of rats. We show that differences in fluid volume can be tracked both behaviorally and neurophysiologically while ensuring that no major electrical noise coming from the device would affect neurophysiological recordings.

### Comparison with commercial syringe pump

In an effort to compare the functionality of our syringe pump controller setup against a current commercial syringe pump, we performed electrophysiological recordings while activating a syringe pump 100 times for 300 ms across a 2 min test session. A headstage cable was plugged into a headstage testing board (HTB_10_, Plexon), which is a passive breakout board used to troubleshoot data acquisition systems. We recorded “noise” from the breakout board for 2 min while syringe pumps were activated. This was performed with our syringe pump controller and with a commercial syringe pump (Single Speed Syringe Pump, PHM-100, Med Associates). Additionally, we tested the commercial syringe pump again with incorporating a resistor-capacitor (RC) “snubber,” which is a device that suppresses voltage transients. A Quencharc Arc Suppressor (Snubber Network Metallized Polyester Film Capacitor, Cornell Dubilier Electronics) was placed inside the commercial syringe pump with the leads of the snubber attached to the two ground leads on the motor inside the syringe pump.

### Calibration methods

A 50 ml volumetric flask and a scale with 0.1 μg resolution was used to obtain the density of distilled water. A 22 gauge dispensing needle was attached to the syringe. A 30 or 60 ml (BD) syringe was attached to the pump and primed with fluid before data collection. Liquid was dispensed in 500 step intervals for the 30 ml syringe and 400 step intervals for the 60 ml syringe. The mass of each dispensing interval and their averages were recorded and are available in the spreadsheet in Extended Data 1.

### Experiment validation methods

#### Animals

All animal procedures were performed in accordance with the regulations of the American University animal care committee. Five male Long–Evans rats were used to validate the functionality of the syringe pump controller paired with electrophysiological recordings. Rats were implanted with a 2 × 8 multielectrode array in the medial frontal cortex (anteroposterior, +3.2; mediolateral, +1.0; dorsoventral, −1.5). Local field potential (LFP) activity was recorded using a Plexon Multichannel Acquisition Processor (Plexon).

#### Task

Rats were trained in a shifting values licking task. Briefly, rats must lick at a spout to receive a liquid sucrose reward. Typically, rats receive alternating concentrations of liquid sucrose rewards every 30 s at a single drinking spout in the behavior box. Here, instead of alternating the concentration of liquid sucrose rewards, the reward volume was changed. Rats received a 16% (wt/vol) concentration of sucrose, while the volume of the reward would shift from a large (∼30 μl; 27.85 μl exactly) to a small (∼10 μl; 9.284 μl exactly) reward. The timing of the reward was set at 0.5 s for both volumes. A single lick at the spout would trigger the syringe pump controller to deliver one of two fluid volumes. Subsequent licks during the fluid delivery time do not “retrigger” the fluid delivery, but instead the first lick after fluid delivery would trigger the next pump activation.

#### Behavioral apparatus

Plexiglas operant chambers (Med Associates) contained a glass drinking spout that was centered on one side of the operant chamber wall at a height of 5 cm from the chamber floor. Tygon tubing connected to the back of the drinking spout would administer the fluid from a 60 ml syringe (BD) hooked up to the syringe in the syringe pump holder. A “light-pipe” lickometer (Med Associates) detected licks via an LED photobeam, and a lick would trigger the syringe pump to deliver one of the three possible fluid volumes via a falling edge transition input to the pump controller. Behavioral protocols were run though Med-PC version IV (Med Associates), and behavioral data were sent via Med Associates superport card TTL (transistor-transistor logic) pulses from the Med-PC software to the syringe pump and subsequently to the Plexon recording system via a solid-state relay.

#### Data analysis

Data were analyzed in NeuroExplorer (LFP, power spectra), MATLAB, and Python (lick data). The Python scipy statistics package was used to perform Wilcoxon rank sum tests for the interlick intervals of each rat.

#### Code accessibility

The code described in the article is freely available on-line on GitHub https://github.com/LaubachLab. The code is also available in Extended Data 1.

## Results

The syringe pump controller delivers different volumes all set to a common time scale. This is accomplished through algorithm development based on simple physics. Validation of fluid volume delivered for both distilled water and sucrose solutions of 30 and 60 ml syringes can be found in a spreadsheet in Extended Data 1. After testing and measuring fluid delivery 36 times at 400 steps for a 60 ml syringe (500 steps for a 30 ml syringe), the average fluid delivery for each step of the motor when using distilled water is 1.88 μl (SD = 4.86 × 10^−5^) for a 60 ml syringe, and 1.24 μl (SD = 1.35 × 10^−5^) for a 30 ml syringe.

Additionally, the pump can functionally be used when paired with electrophysiological recordings of LFP activity in awake, behaving rats. Rats were tested in a shifting values licking task with 30 s shifts of access to a large (∼30 μl reward; 27.85 μl exactly, with setting p3 = 15 in our setup) volume reward and a small (∼10 μl; 9.28 μl exactly, with setting p1 = 5) volume reward of 16% sucrose. The large reward was configured to be exactly three times the size of the small reward.

**Figure 4.**
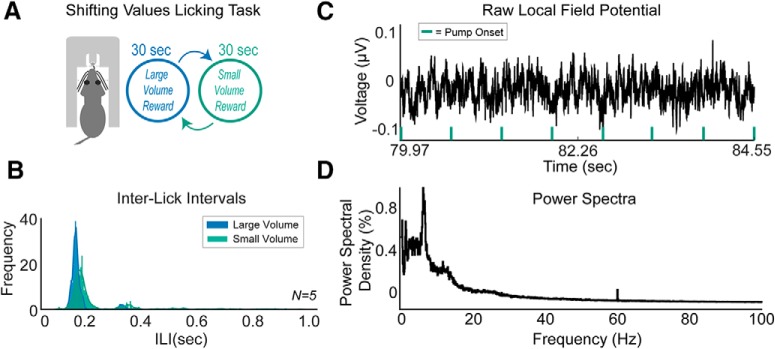
Validation in behavioral experiment. ***A***, The syringe pump controller was validated by using it in a shifting values licking task in a group of rats (*N* = 5). Rats lick at a spout to receive a liquid sucrose reward. Reward was delivered for 0.5 s and would switch every 30 s from a large-volume (∼30 μl) to a small-volume (∼10 μl) reward. ***B***, Rats licked differently for large-volume versus small-volume rewards. Interlick intervals of all five rats were increased for small-volume rewards. ***C***, Raw local field potential activity revealed no major differences during the syringe pump onset. ***D***, Power spectra of the local field potential activity of one rat showed no major electrical noise at 60 Hz (under 0.2% of the 30 min session had 60 Hz electrical noise).

Figure 4 shows licking behavior (interlick intervals) from a group of five rats as they participated in the shifting values licking task (Fig. 4*A*). A lick to the spout would activate either the large or small reward for 0.5 s. Rats licked differently for large versus small volume rewards of 16% liquid sucrose (Fig. 4*B*); more details on these behavioral and electrophysiological results will be published in a forthcoming manuscript (L. Amarante and M. Laubach, unpublished observations). Notably, rats licked at higher frequencies when consuming the larger volume reward, as evidenced by Wilcoxon tests for the interlick intervals of each rat during large and small volume fluid consumption (*p* < 0.0001 for all rats).

**Figure 5.**
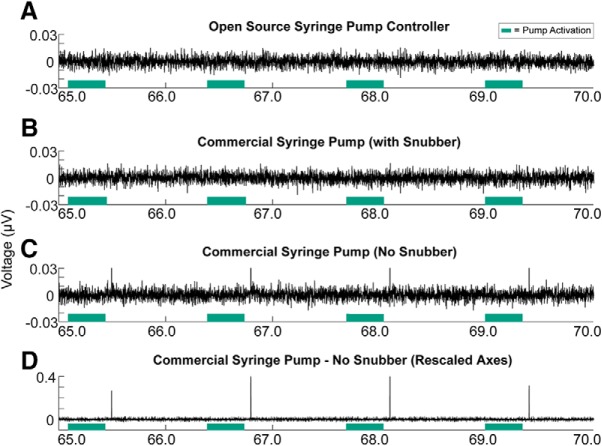
Comparison of open source syringe pump controller against a commercial syringe pump in electrophysiological recordings. ***A***, Electrophysiological recordings were made for 2 min, while the open source syringe pump controller was activated 100 times for 300 ms. The open source syringe pump controller did not produce any electrical transients, similar to in Figure 4*C*. ***B***, When testing a standard commercial syringe pump in the same manner, electrical transients (called shot noise) were apparent within 100 ms after the syringe pump turned off. ***C***, A zoomed-out view with rescaled axes shows the electrical transients across 5 s of recordings. ***D***, An RC snubber/arc suppressor was added into the commercial syringe pump, which effectively suppressed transients. Green boxes designate when the syringe pump was activated with the motor running.

Raw LFP traces in the medial frontal cortex of rats did not show any major fluctuations in electrical noise when the syringe pump was activated (Fig. 4*C*), and a power spectral density graph did not show any major increase in 60 Hz electrical noise across the session (Fig. 4*D*; <0.2% of the session consisted of 60 Hz). Notably, when compared against a standard commercial syringe pump, our syringe pump controller performed optimally in that it did not produce electrical voltage transients (Fig. 5*A*), whereas the commercial syringe pump reliably gave off electrical transients just after the syringe pump turned off (Fig. 5*C*,*D*). To prevent this, the same commercial syringe pump was tested again, but with a Quencharc Arc Suppressor snubber placed inside and connected to the motor of the pump. This device effectively blocked electrical transients, allowing the commercial syringe pump to function properly for use with electrophysiological recordings (Fig. 5*B*).

## Discussion

We have developed an open source syringe pump control circuit that delivers fluid rewards of different sizes across one common time epoch from one single syringe. There are many benefits to using this syringe pump including low cost (under $250 including the syringe pump controller and 3D printed pump holder), ease of assembly, ease of customization, delivery of multiple fluid volumes delivered on a microliter scale, the ability to dissociate the confound of time and reward size, and the ability to use the pump with electrophysiological recordings. As an alternative, we would like to point out that a solenoid-based design exists (Mitz, 2005), which is apparently available commercially. This device is ideal for the issues addressed here and is capable of delivering smaller precise fluid volumes. However, the project was written up more as a report on the capabilities of the system and did not include complete build instructions.

The control circuit can be attached to any stepper motor or external syringe pump holder. For accuracy, we used a calibrated stand-alone NEMA 17 integrated stepper motor + driver (Applied Motion Products), but other bipolar stepper motors can also be used with this setup. The device is all open source, and PCB designs can easily be reprinted to replicate the controller. The control board could be implemented into behavioral paradigms quite simply, as it only needs a voltage input to trigger fluid delivery. We validated the syringe pump controller by using it in a shifting values licking task with two different reward volumes. In its default form, the syringe pump controller can deliver up to three volumes and could potentially be set up for more inputs if the code is adjusted by the user.

Through using a 30 ml syringe, we were able to dispense 1.24 μl of fluid per one single motor step. Smaller volumes of fluid may be able to be obtained through using a smaller (10 ml) syringe, though it is difficult to tell whether the animal would actually be receiving the entire bolus of fluid with a reward under 1 μl. Additional caveats that would potentially degrade the accuracy of the volume dispensed would be the setup of the syringe to a spout (flexibility, length, or orientation of the tubing connecting the syringe to the drinking spout), and it is possible that a glass syringe (instead of a plastic syringe) would yield the most accurate fluid delivery. It is important to keep in mind that a different diameter syringe (e.g., a 60 ml syringe from BD vs a 60 ml syringe from MedLine have different inner diameters by ∼2 mm) would also yield different volumes, and therefore it is necessary to perform a syringe pump calibration for the setup of each individual laboratory. It is important to note that this syringe pump controller is intended for its functionality in the delivery of multiple fluid volumes. Other syringe pump controllers (https://github.com/wgpatrick/opensyringepump/) may be sought out if submicroliter precision is desired.

We compared the functionality of our open source syringe pump controller with a commercial syringe pump and found that the commercial syringe pump produced significant electrical transients, or “shot noise,” just after the pump would turn off. Our open source syringe pump did not have any hint of electrical transients and makes it optimal to combine with electrophysiological recordings. We would like to note that adding an RC snubber/arc suppressor to the commercial syringe pump did effectively suppress electrical transients, as this may be a useful tip to laboratories that are already using a commercial syringe pump in their experiments or are looking to design their own syringe pump controller.

The control circuit is novel to the neuroscience field since it can be used to reassess classic neuroscience studies that have confounded timing and reward delivery, for example by leaving a solenoid valve open longer to deliver a larger reward. A major benefit of using this control circuit is the implementation of the equation within the code to adjust the velocity and acceleration of the motor to provide equal timing of different volume rewards. This reduces the confound between time and reward size, which has been a potential issue of previous studies on reward magnitude. With this device, it will be possible to assess whether “reward signals” in electrophysiological data are due to the timing or size of the reward. Additionally, the device will allow for at least three different volumes to be dispensed, making it possible for future studies to investigate reward size and comparison of multiple rewards. The device minimizes the need for multiple syringe pumps or devices by having just one single syringe deliver different volumes of reward. The syringe pump controller opens the door to new studies that aim to manipulate reward size using one single device.

## Synthesis

Reviewing Editor: Yavin Shaham, NIDA-IRP/NIH Decisions are customarily a result of the Reviewing Editor and the peer reviewers coming together and discussing their recommendations until a consensus is reached. When revisions are invited, a fact-based synthesis statement explaining their decision and outlining what is needed to prepare a revision will be listed below. The following reviewer(s) agreed to reveal their identity: Matthew Gardner.

The reviewers found significant merit in the study but have raised several minor concerns that should be addressed in the revision. I enclose below the individual comments of the reviewers and below each of them my recommendation of how to address these comments. In addition to the reviewers' comments, please address the minor comments below:

-1. Introduction: change “To investigate this issue” to “To address this issue”

-2. Page 9: change “most commonly” to “commonly”

REVIEWER 1

The current report describes the design and implementation of an open source syringe pump controller that is capable of delivering three preset reward volumes during a fixed delivery duration. The system will allow researchers to better dissociate reward value and reward timing in tasks that employ multiple rewards to study reward processing.

The paper is well written and the authors provide detailed instructions for device assembly and use. While the controller currently performs a very specific function - modulate fluid volume while keeping delivery time constant - the open-source nature of the project will undoubtedly enable other adaptations in the future.

I have a few minor suggestions to improve readability and replication:

1. I would recommend that the authors include a 3D rendering of the complete setup including the controller, pump, syringe holder and power supply as part of figure 1. If possible, they could provide an exploded view of this setup and highlight which pieces they redesigned etc. This rendering could also serve as their graphical abstract. I would also suggest that they increase font sizes in figure 2 to improve readability and shift pin labels out of the board in D (similar to C).

EDITOR: I suggest adding this feature to the revised version.

2. It would be beneficial to show a plot of predicted vs.measured volumes delivered by the controller when used in their experiment. The current calibration plot only uses a fixed step size. It would be nice to see how the system performs when switching between volumes delivered (eg. in Figure 3)

EDITOR: Please add this plot and data to the revised version.

3. The authors state that their system shows ‘no major electrical noise’. It would be beneficial to show a comparison of noise spectra between the current design and a commercial pump system with single speed capability.

EDITOR: If possible, please add this comparison to the revision but addressing this reviewer's comment is not mandatory for a successful revision.

Overall the current study provides neuroscientists with a low-cost tool for variable volume fluid delivery and guides the reader through the steps required to build this device. I commend the authors for their clear description and detailed supplementary materials. I look forward to their forthcoming data paper.

REVIEWER 2

The authors provide an open source design for a syringe pump with the primary use being for fluid delivery in animal behavior experiments. The syringe pump has the capability of delivering fluids at various rates in order to deliver different amounts of fluid over the same amount of time; a capability which is not common among commercially available syringe pumps and is useful for electrophysiology experiments. The cost of the open source pump appears to be substantially lower than a commercial pump, and the authors state that the pump has minimal electrical noise associated with it - certainly a plus for electrophysiology experiments.

I am happy to see that the authors have taken their time to organize and publicly share the details of the pump they have assembled. It is a relatively common issue with animal behavior to not have quite the ideal apparatus for a particular experiment, and the more DIY solutions that are shared publicly I believe will in general help to improve the field. The ability to deliver different fluid amounts over the same time period is an important advance which will help to disambiguate sensory signals from temporal coding in neural processing. Certainly, as the authors point out, this is a common confound which has been overlooked in many electrophysiology studies.

I think the authors have done more than enough to describe their device in the manuscript, and I will assume the Github link has reasonable documentation to assemble and get the device working (the Github url is anonymized for the reviewer).

I have just a few minor questions which I think could help to better clarify the manuscript.

1. I was a bit confused by the ‘how to change reward timing section’. At first, it seemed that you simply just enter the number of steps for the different reward amounts: p1, p2, and p3 and t is the time of the delivery. Yet, the paragraph then suggests that the acceleration must be adjusted in the firmware, and that p3 is now somehow different from p1 and p2. The confusion is likely due to this reviewer's failure to follow the text, but perhaps the authors could unpack this paragraph a bit more?

EDITOR: please address the reviewer's comment with text revisions.

2. I was surprised to see a mix of voltages for the digital IO. I think the authors state that the output that keeps track of the pump being on is a 3.3 volt TTL signal and that the input takes a 0-5 volt TTL. I think its uncommon to have a mix of voltages for the TTL signals, it might help to mention why this appears to be the case.

EDITOR: please address the reviewer's comment in the revision.

## Author Response

---------------------------------------------

The reviewers found significant merit in the study but have raised several minor concerns that should be addressed in the revision. I enclose below the individual comments of the reviewers and below each of them my recommendation of how to address these comments. In addition to the reviewers' comments, please address the minor comments below:

-1. Introduction: change “To investigate this issue” to “To address this issue”

-2. Page 9: change “most commonly” to “commonly”

We have made these changes, and have highlighted these edits along with some additional typos we found, in red throughout the document.

Additionally, we removed the green schematic of the printed circuit board that was previously in figure 1, as it was largely redundant to the blue/black PCB schematic in figure 1.

REVIEWER 1

The current report describes the design and implementation of an open source syringe pump controller that is capable of delivering three preset reward volumes during a fixed delivery duration. The system will allow researchers to better dissociate reward value and reward timing in tasks that employ multiple rewards to study reward processing.

The paper is well written and the authors provide detailed instructions for device assembly and use. While the controller currently performs a very specific function - modulate fluid volume while keeping delivery time constant - the open-source nature of the project will undoubtedly enable other adaptations in the future.

I have a few minor suggestions to improve readability and replication:

1. I would recommend that the authors include a 3D rendering of the complete setup including the controller, pump, syringe holder and power supply as part of figure 1. If possible, they could provide an exploded view of this setup and highlight which pieces they redesigned etc. This rendering could also serve as their graphical abstract. I would also suggest that they increase font sizes in figure 2 to improve readability and shift pin labels out of the board in D (similar to C).

EDITOR: I suggest adding this feature to the revised version.

We agree that a full view of the setup should be included. Adding in a 3D rendering of the entire device would require quite a bit of time and effort as well as time and training for us to learn how to create some of the parts. Instead, we have provided a full view of the entire setup as a new Figure 2, which includes our 3D printed syringe pump holder with the integrated motor + driver attached, the controller itself, and the 15 V power supply used. We have added onto our Github the .stl files that have been redesigned along with a table explaining which parts have been redesigned.

Additionally, we edited the board and teensy pin out panels (Figure 2 C,D) to increase font size and we moved the labels to the outer parts of the board for readability.

2. It would be beneficial to show a plot of predicted vs.measured volumes delivered by the controller when used in their experiment. The current calibration plot only uses a fixed step size. It would be nice to see how the system performs when switching between volumes delivered (eg. in Figure 3)

EDITOR: Please add this plot and data to the revised version.

It is difficult to include a plot or data like this, considering there are no “predicted” versus “measured” volumes; instead, reward volumes need to be calibrated by the specific user. This system requires the user to calibrate the volumes based on their individual lab's setup. For example, using a different type of syringe (glass versus plastic, or 30mL versus 60mL) or using a different viscosity of fluid could potentially change the fluid volume dispensed for a given “step” setting. Therefore, we insist throughout the manuscript (As stated in the Velocity Profile Algorithm section of the methods, “The amount of fluid delivered per step must be determined via a calibration procedure for a particular syringe design as detailed below.” This is also stated in the discussion: “and therefore it is necessary to perform a syringe pump calibration for each individual lab's setup”) that the user performs the calibration methods to identify the fluid volume desired for their experiment, and then use those corresponding step values for p1,p2, and p3 in their experiments. We emphasized this by including the calibration methods as the first step in the methods section “Basic Instructions for Operation”. We also emphasized this in the text on the relevance of using the NEMA-17 integrated stepper motor + driver instead of the cheaper NEMA-17 motors which would not provide as reliable motor rotations; therefore, once a volume calibration is performed, these values should hold true for a while, with the user only needing to check for accurate volume output periodically.

3. The authors state that their system shows ‘no major electrical noise’. It would be beneficial to show a comparison of noise spectra between the current design and a commercial pump system with single speed capability.

EDITOR: If possible, please add this comparison to the revision but addressing this reviewer's comment is not mandatory for a successful revision.

We think this is a great idea and only improves the manuscript to compare our controller to commercial syringe pumps. To provide some background, our initial intent for creating our own “noise-less” syringe pump is because the commercial pump we use for behavioral experiments (from Med Associates) produces a large amount of electrical shot noise / electrical transients when activated that inundate LFP or spike recordings.

Our “quick fix” in the lab was to purchase resistor-capacitor RC snubbers, which are devices that suppress electrical voltage transients. However, this requires us to open up each individual commercial pump and place the snubber devices inside the pumps, which is an added extra step when already paying over 500 dollars for the commercial syringe pump (and that pump specifically still can only deliver one rate of fluid volume). We therefore have added into the figure four voltage traces from recordings; one with our syringe pump controller activated 4 times across 5 seconds, then with the commercial syringe pump as it would arrive straight from the company (and a zoomed in image showing the transients on a rescaled axis), and then finally with the commercial syringe pump with the RC snubber incorporated into the pump.

These recordings are of “noise”, with a headstage cable attached to a Plexon headstage test board (HTB-10, Plexon, Inc; Dallas, TX), and with the test board placed inside the behavior box next to the fluid delivery spout. We've added this data as its own figure and added additional paragraph in the methods section on comparing the pumps. We added a paragraph about this in the discussion again to emphasize the caution in using the commercial pumps with electrophysiology, and also to provide the tip to others to include the RC snubber into their devices.

Overall the current study provides neuroscientists with a low-cost tool for variable volume fluid delivery and guides the reader through the steps required to build this device. I commend the authors for their clear description and detailed supplementary materials. I look forward to their forthcoming data paper.

REVIEWER 2

The authors provide an open source design for a syringe pump with the primary use being for fluid delivery in animal behavior experiments. The syringe pump has the capability of delivering fluids at various rates in order to deliver different amounts of fluid over the same amount of time; a capability which is not common among commercially available syringe pumps and is useful for electrophysiology experiments. The cost of the open source pump appears to be substantially lower than a commercial pump, and the authors state that the pump has minimal electrical noise associated with it - certainly a plus for electrophysiology experiments.

I am happy to see that the authors have taken their time to organize and publicly share the details of the pump they have assembled. It is a relatively common issue with animal behavior to not have quite the ideal apparatus for a particular experiment, and the more DIY solutions that are shared publicly I believe will in general help to improve the field. The ability to deliver different fluid amounts over the same time period is an important advance which will help to disambiguate sensory signals from temporal coding in neural processing. Certainly, as the authors point out, this is a common confound which has been overlooked in many electrophysiology studies.

I think the authors have done more than enough to describe their device in the manuscript, and I will assume the Github link has reasonable documentation to assemble and get the device working (the Github url is anonymized for the reviewer).

I have just a few minor questions which I think could help to better clarify the manuscript.

1. I was a bit confused by the ‘how to change reward timing section’. At first, it seemed that you simply just enter the number of steps for the different reward amounts: p1, p2, and p3 and t is the time of the delivery. Yet, the paragraph then suggests that the acceleration must be adjusted in the firmware, and that p3 is now somehow different from p1 and p2. The confusion is likely due to this reviewer's failure to follow the text, but perhaps the authors could unpack this paragraph a bit more?

EDITOR: please address the reviewer's comment with text revisions.

We agree that it was not entirely clear to readers in this section and we have since reworded this section. Our intent was to emphasize that the value of p3 will determine the time scale variable (t), so we have clarified this in the text.

2. I was surprised to see a mix of voltages for the digital IO. I think the authors state that the output that keeps track of the pump being on is a 3.3 volt TTL signal and that the input takes a 0-5 volt TTL. I think its uncommon to have a mix of voltages for the TTL signals, it might help to mention why this appears to be the case.

EDITOR: please address the reviewer's comment in the revision.

Thank you for this comment; we recognize that our description of the TTL outputs were not entirely clear. To clarify, the input pins to the control board take a falling edge voltage transition, meaning that these pins (pin 18, 19, and 20) can take up to a 5V input but the device will recognize an input that brings the pin from 5V down to 0V. A Teensy 3.2 microcontroller has 5V tolerance, meaning it can take up to a 5V input. Our intent is to emphasize that it is a falling edge voltage transition that will be recognized, meaning that the pins can take any input that is under 5V to trigger the fluid output.

The 3.3 volts is not an input but rather the output from the Teensy 3.2 which can innately output up to 3.3V. Therefore, the LED attached the pin 23 is turned on / will light up when voltage on the teensy pin goes “HIGH” (goes from 0V to 3.3V).

We have clarified our description of this in the methods section of “How to connect to behavioral equipment” by removing details on the 5V input and instead referring to the falling edge transition, since any decrease in voltage will be read as an input.
