# Supplementary material for: An Open Source Syringe Pump Controller for Fluid Delivery of Multiple Volumes
Source: eNeuro. 2019 Sep 6;6(5):ENEURO.0240-19.2019. doi: 10.1523/ENEURO.0240-19.2019 (PMC6734045; doi:10.1523/ENEURO.0240-19.2019)

Open Source Syringe Pump Build Instructions

These instructions require basic understanding of electronics assembly and programming techniques. In addition to a temperature regulated soldering iron and solder, it is recommended that these instructions be followed in the neighborhood of a good pair of pliers, wire cutters, wire strippers and fresh desoldering wick.

Connecting to and Programming the Teensy 3.2 Microcontroller

1. Connect the Teensy microcontroller to a computer:
    a. Follow the Teensyduino installation instructions. This requires that you install the Arduino application and then the Teensyduino application on top of Arduino.
    b. Connect the Teensy 3.2 to a computer using a micro-usb cable. Be gentle with the micro-usb connector on the Teensy 3.2 as it is not terribly robust.
    c. When connected, the Teensy should enumerate as a USB device and commence blinking its orange LED once every two seconds.
2. Program the Teensy 3.2 microcontroller:
    a. Open the Arduino application and ensure that you have selected:
        i. Tools→Board→Teensy 3.2/3.1
        ii. Tools→USB Type→Serial
        iii. Tools→CPU Speed→96 MHz (overclock)
        iv. Tools→Optimize→Faster
        v. Tools→Port→[your Teensy port]
    b. Navigate to File→Examples→01.Basics→Blink
    c. Find the two lines of code that read “delay(1000);” Modify these lines so that each reads “delay(250);”
    d. Upload the code by navigating to Sketch→Upload, or click on the upload hotkey . This should compile the modified “Blink” sketch to the Teensy and blink the orange LED at a faster rate.
    e. Congratulations, you successfully programmed the Teensy microcontroller!

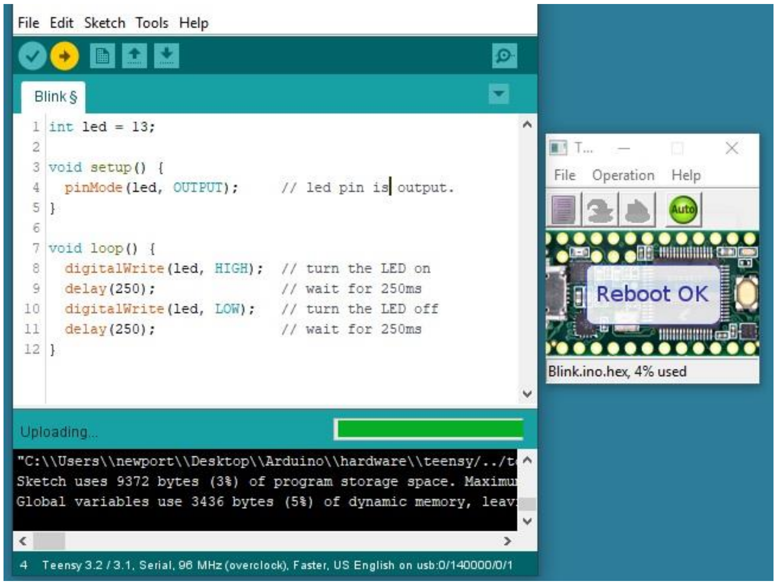

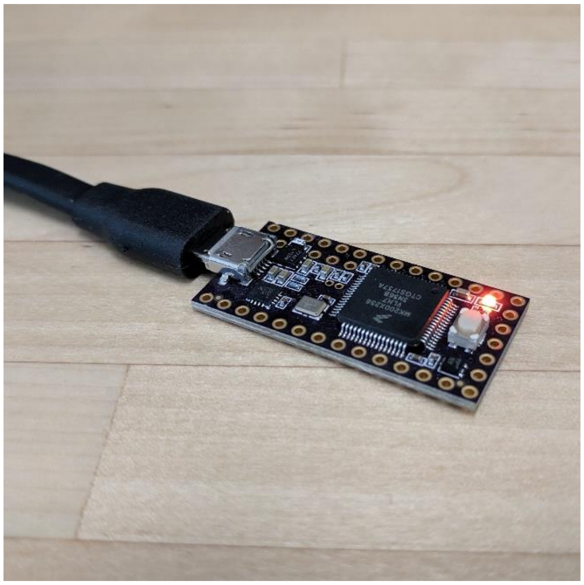

Teensy and TeensyView Soldering and Installation

3. Locate a length of extra-long headers with 0.1” spacing break off 14-pin, 13-pin and 2-pin lengths. Arrange them in a solderless breadboard so that the exposed tops are even and the unsoldered Teensy board fits over all pins as shown in the pictures below.
4. Proceed to solder the header pins to the Teensy through-hole pads.
    a. Soldering notes: heat both the pin and the pad with the soldering iron on one side of the pin and apply a small amount of solder to the opposite side of the pin. Hold the tip of the soldering iron on the pin and the pad until you see solder drawn into the interstitial space between the header pin and the plated through hole. It is beneficial to keep the solder off the “stalk” of the header pin, as the TeensyView display module needs to slide on these headers.

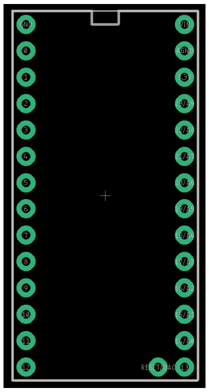
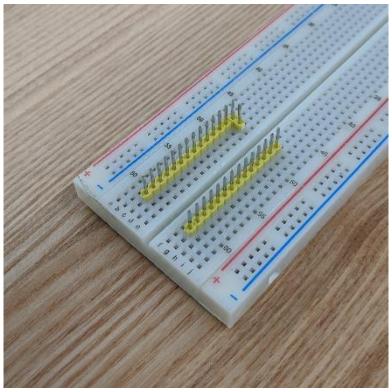
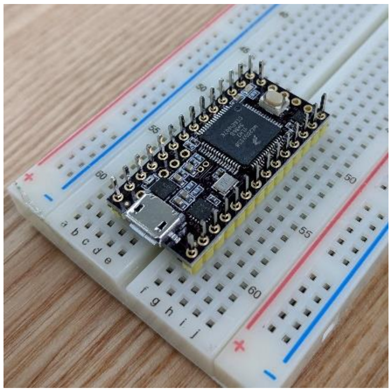
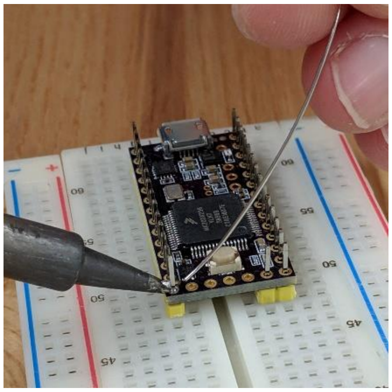

5. After all header pins are soldered, use a flush cutter to trim the A14/DAC pin from the top of the board.
6. Break off two 14-pin lengths of female headers and place them on the Teensy along with the TeensyView OLED display. You need only to solder those pins which are circled on the TeensyView board; however, soldering all pins provides you an opportunity to hone your soldering skills.

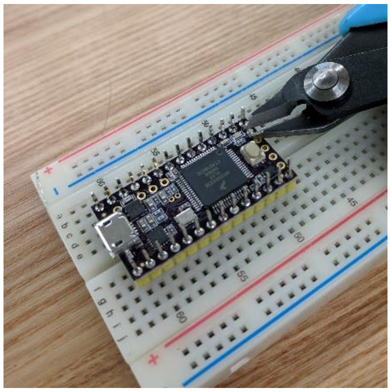
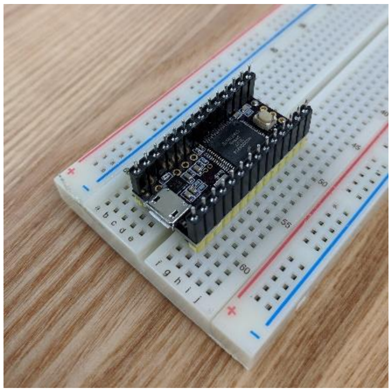
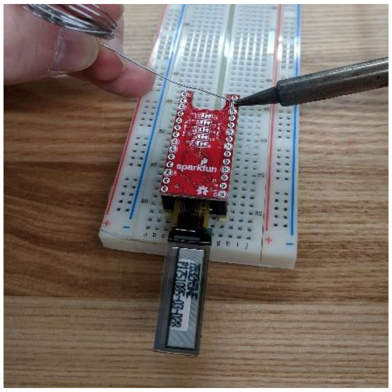

7. Cut two small pieces of the provided double-sided foam tape and apply to the back of the OLED display. Peel the remaining adhesive protectant from the tape and carefully position the OLED display on the TeensyView carrier board.

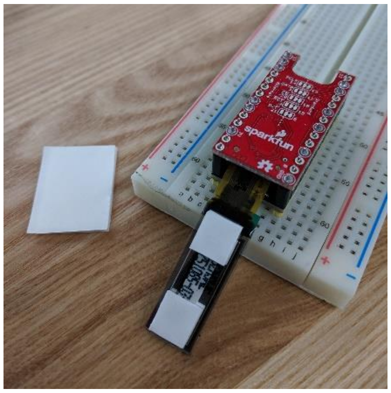
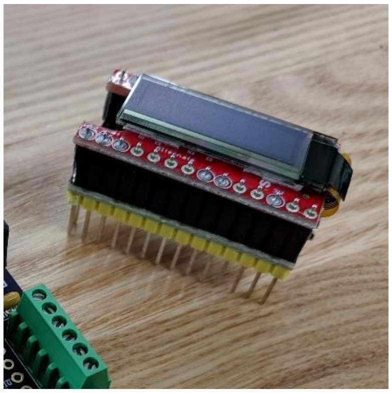
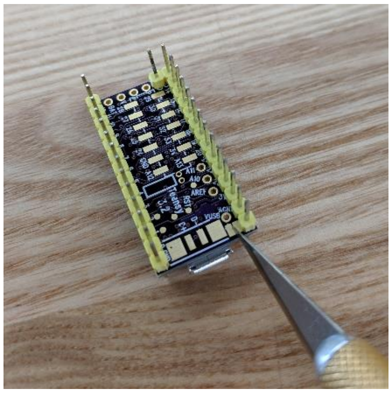

Test the TeensyView Display

8. It is suggested that you reconnect the Teensy to a computer and ensure the "Blink" sketch still works. If it does not, carefully inspect all solder joints and repair any bridges.
9. Install the TeensyView Library:
    a. Open Arduino and Navigate to Sketch→Include Library→Manage Libraries…
    b. In the search box, type "teensyview".
    c. This should yield a single result, "TeensyView by SparkFun Electronics". Install this library and close the Library Manager.
    d. Navigate to File→Examples→TeensyView→ScreenDemo and upload this code to the Teensy microcontroller.
    e. Revel in your burgeoning ability to control the pixels of an Organic Light Emitting Diode Display.

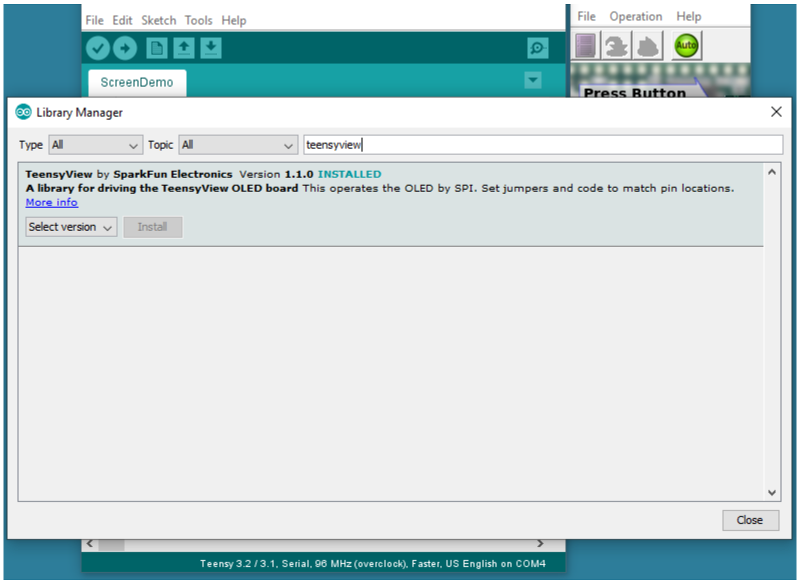

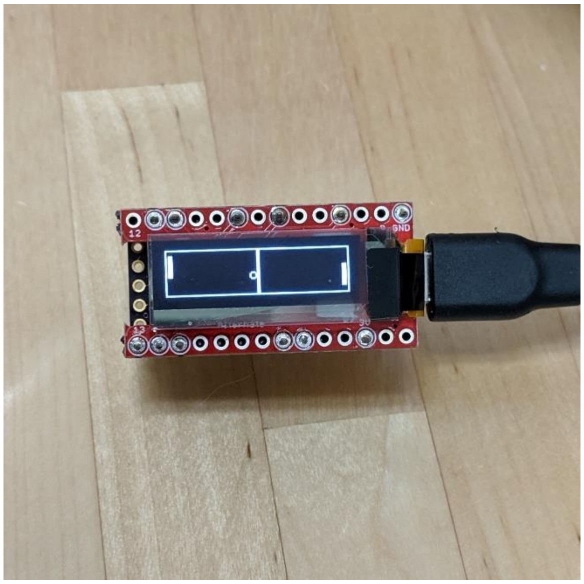

Solder Components onto the Printed Circuit Board

1. Though you may solder the components onto the board in any order, in general it is easiest to solder components that are low to the board first, then progressively solder taller and taller components.
2. Start by inserting the 100kΩ resistor **R1** (with color code Brown-Black-Yellow), pushbutton switches, 1µF capacitors **C2** and **C3**, and 10V Zener diode **D1**. Note that the Zener diode is directional and must be placed so that the wire nearest the black band is inserted in the hole nearest the white band on the board (as shown below). The board will not operate if the diode is installed backwards.
3. There are a few ways to keep the components near the board whilst soldering the backside of the board. The simplest is to bend the leads to keep components seated on the board as you solder. High temperature tapes such as Kapton polyamide or powder-coat tape can be used to secure components. In some circumstances, you might use your fingers, though at the risk of scorched flesh.

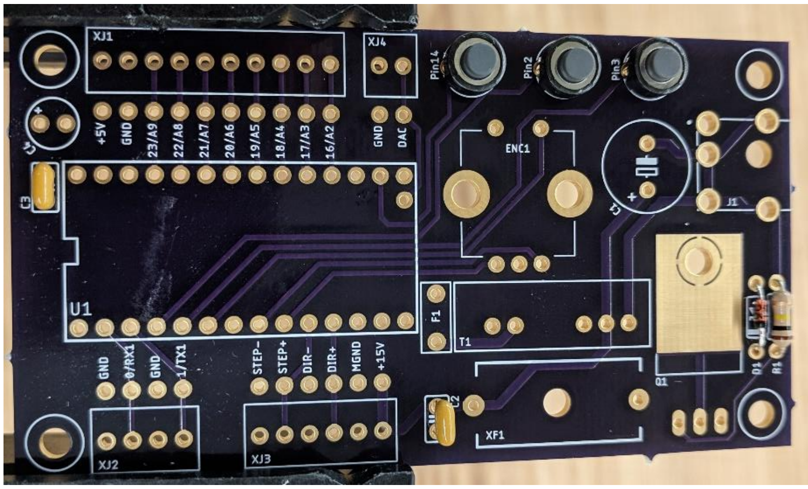

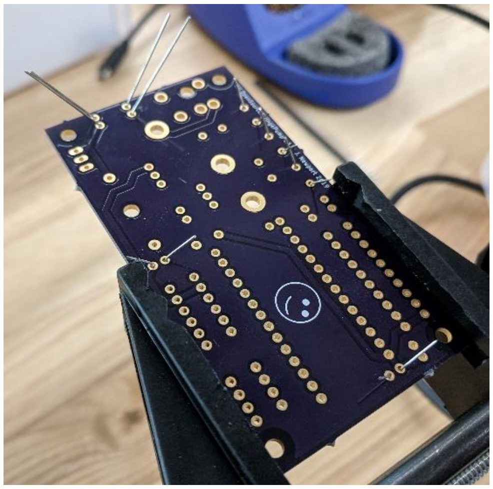

4. Next, install the power connector **J1** and the FQP47P06 MOSFET **Q1**. Though not necessary, the MOSFET may be soldered to the underlying exposed copper or screwed down using an M3 or 4-40 screw and nut. If using metal nuts and/or screws, do not overtighten, as this could dig through the solder mask and into the ground plane of the PCB, rendering the board inoperative.
5. Install the terminal headers. Locations **XJ1**, **XJ2**, **XJ3** and **XJ4** were designed to accept any headers that have 0.1in (2.54mm) component spacing. This includes the recommended screw terminal blocks, screwless terminal blocks or standard pin headers. There are two connections for every signal for permanent soldering or testing purposes.
6. Install and solder the 10µF Tantalum Capacitor **C4** and the 500mA trip Polyfuse **F1**.

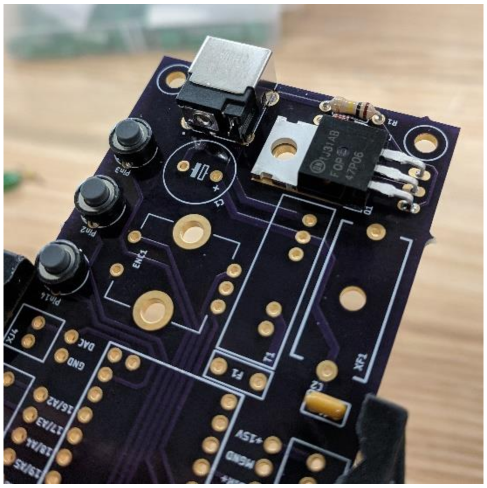
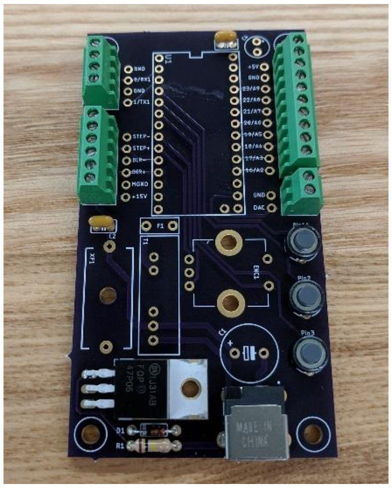
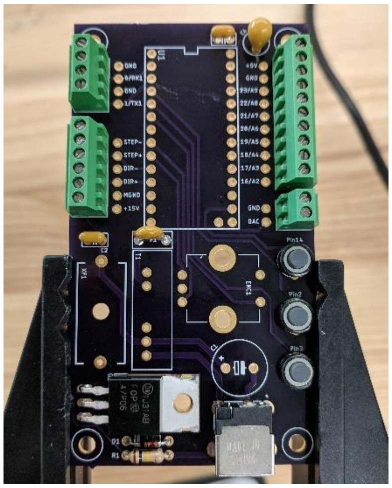

7. Install the DC/DC Converter **T1**. Flatten the leads of fuse holder **XF1** and install. Install the 100µF electrolytic capacitor **C1**, observing polarity. There are large negative signs “-“ on one side of the capacitor. These signs should be facing switch **S2** (or opposite the “+” sign as indicated on the silkscreen). Solder these components in place.
8. Install and solder the rotary encoder **ENC1**.
9. Install the 2A Type 2AG Fast Blow Fuse in the holder. This protects the power supply and the motor from overcurrents.

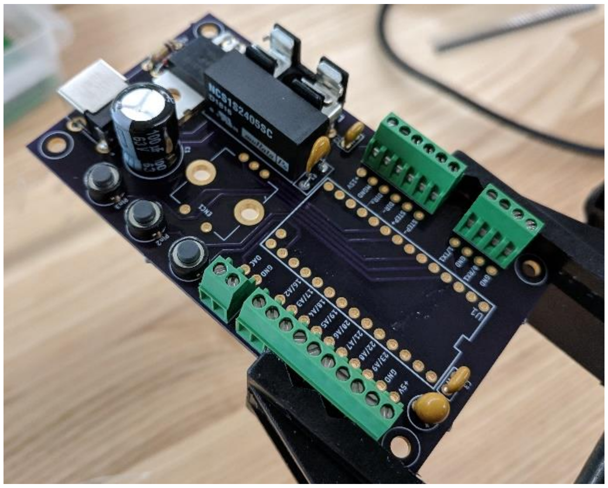
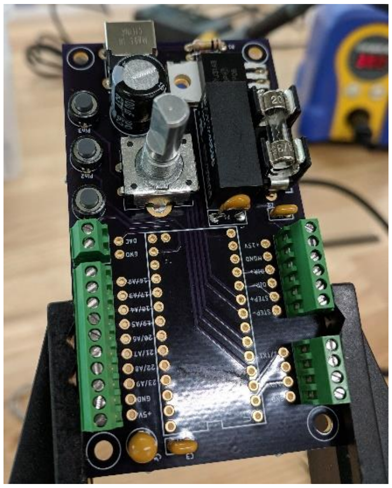

10. It is recommended that you socketize the Teensy microcontroller. Break off lengths of 14, 13 and 2 female 0.1in header pins. Place these in the board and install the Teensy (and optionally the display as well). This will keep the pins aligned as you solder them in.

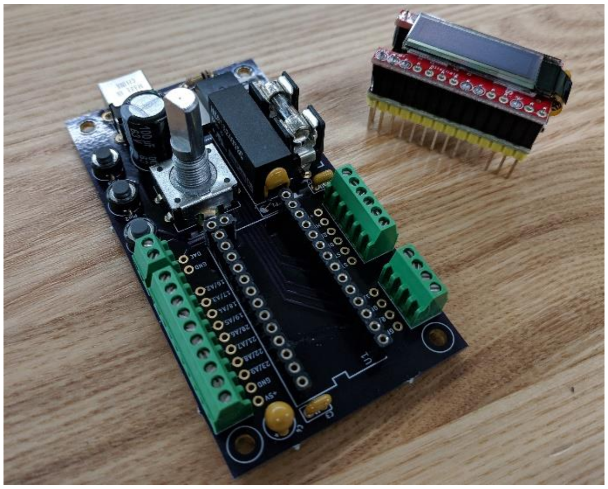
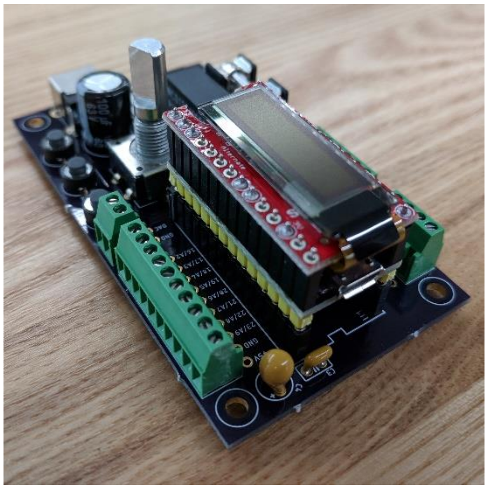

11. Before uploading the firmware to the Teensy, you must cut the VUSB→VIN trace on the backside of the Teensy. This operation separates the attached computer’s power supply from the board’s power supply, allowing for stand-alone operation. After performing this cut, you will not be able to program the Teensy without plugging in the board’s power supply. Flip the teensy over and use a razor knife to cut the trace between the two exposed pads next to the “Vin” pin as shown below. *Note:* If you ever need to undo this operation, simply place a blob of solder between the two pads to reunite them.

12. Install the Teensy in the board and connect the motor's signal cables as labelled. Plug in the power supply.

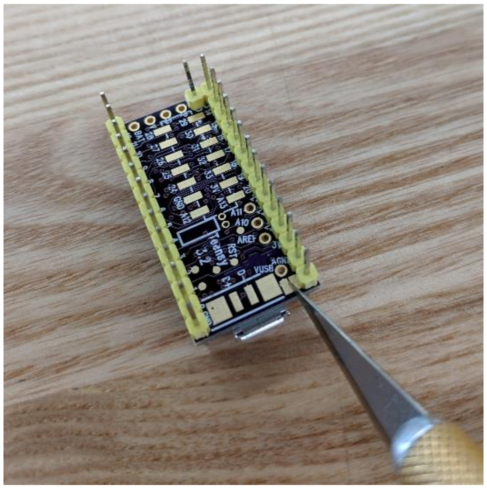

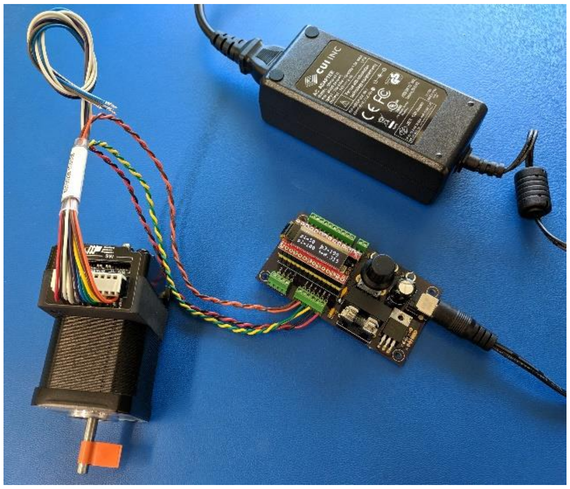

Programming the Teensy Motor Control Firmware and Testing the Motor control Functions

1. It is useful to affix a small piece of tape to the shaft of the motor for testing purposes.
2. The jumper switches located on the Applied Motion stepper motor should be set as shown in the picture below. Refer to the manual to learn more about the function of each of these switches:

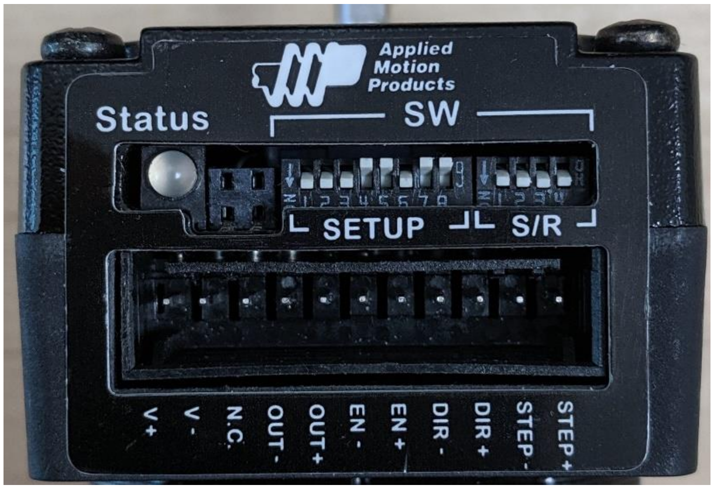

Stepper Controller Options:

1	2	3	4	5	6	7	8
↓	↓	↓	↑	↑	↓	↑	↑

Step Size (Default 200 Steps/revolution):

1	2	3	4
↓	↓	↓	↓

3. Load the "OpenSourceSyringePump.ino" firmware found on github and upload to the Teensy board.
4. The buttons directly above the rotary encoder should run the motor in forward and reverse, and the third button should cycle through a short dance, according to the values on the screen.

Operation

1. Stepper motor rotation is defined by the number of “steps” issued to the motor controller. If the jumper switches are set as shown above, for every 200 pulses issued to the motor via the “STEP+” and “STEP-“ pins, the motor shaft will rotate 360°. If but a single pulse is issued, the shaft will rotate 360°/200=1.8°. The TeensyView screen shows three values (p1, p2 and p3) indicating the number of steps issued to the integrated motor over an equal time period.
2. The values of rotation are activated by a falling voltage transition from 5V to 0V (GND) on pins 18, 19 and 20 which rotates the motor shaft p1, p2 and p3 steps, respectively. Additionally, these step sequences can be activated simply by connecting pins 18, 19 or 20 to GND with a wire or normally open (NO) switch. For example, if p2=100 and pin 19 is connected to GND, the motor will rotate 100 steps, which for the default setup is one-half of a turn or 180°. *Note:* There is a 10kΩ pullup resistor attached to 3.3V internal to the Teensy microcontroller, which also allows any NPN open-collector output to be used as a trigger for the step sequences.

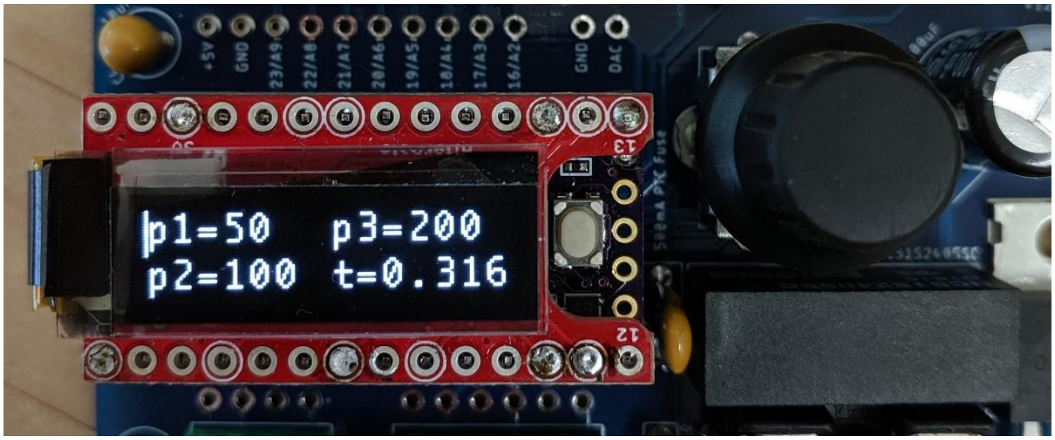

Supplement: Extended Data 1 — Teensy Code Firmware (.ino); PCB design files (.brd, .sch, .pdf) and libraries in Eagle (.lbr); Syringe calibration spreadsheet for water and 16% sucrose (.xlsx); Build instructions (.pdf); Bill of materials (.xlsx); Closeup images of the device (.png). Download Extended Data 1, ZIP file. [file sup_enu-eN-OTM-0240-19-s02.zip › Extended Data/OpenSourceSyringePumpBuildInstructions.pdf]
