# Supplementary figures and images for: An Open Source Syringe Pump Controller for Fluid Delivery of Multiple Volumes
Source: eNeuro. 2019 Sep 6;6(5):ENEURO.0240-19.2019. doi: 10.1523/ENEURO.0240-19.2019 (PMC6734045; doi:10.1523/ENEURO.0240-19.2019)

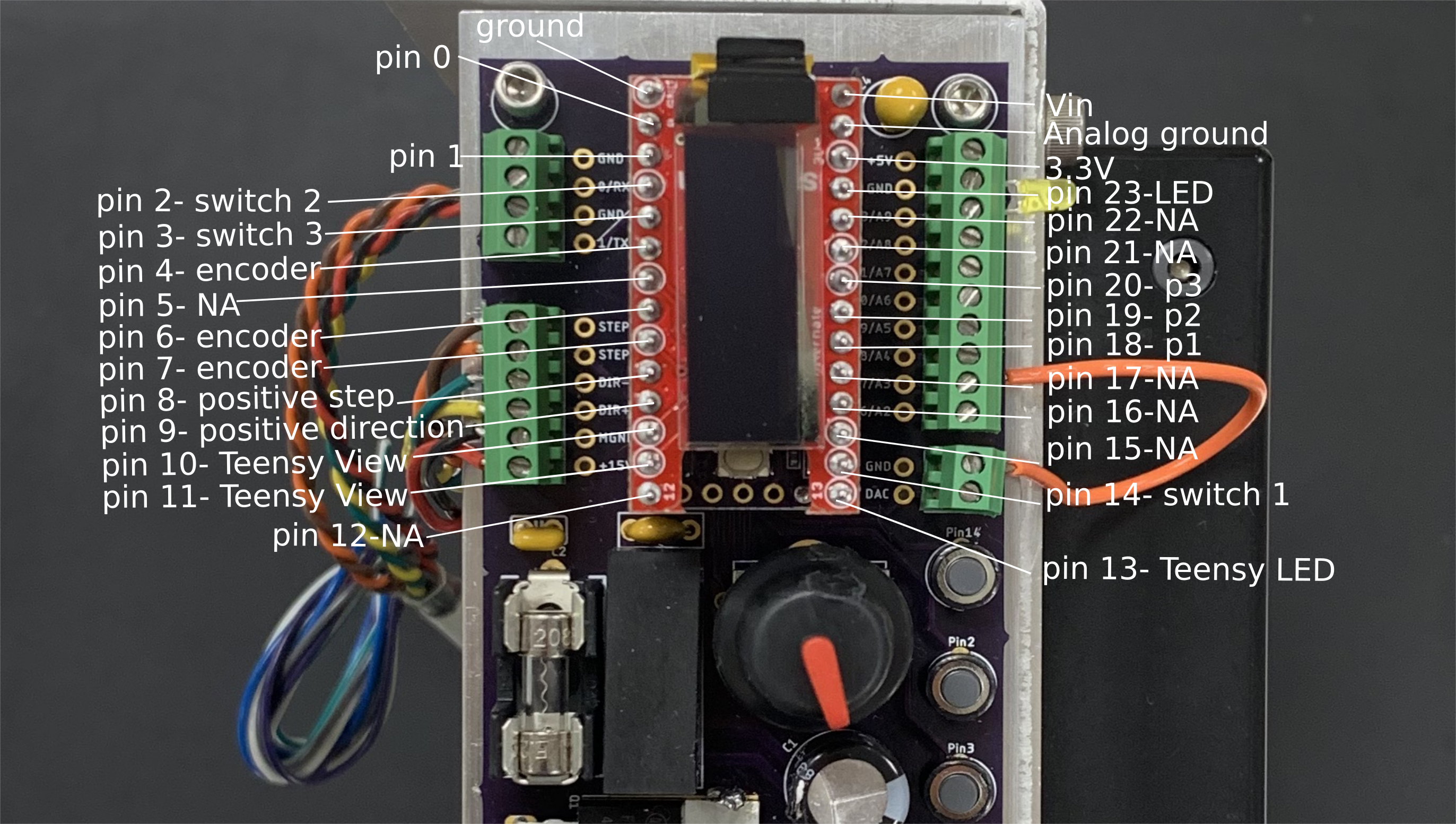

Supplement: Extended Data 1 — Teensy Code Firmware (.ino); PCB design files (.brd, .sch, .pdf) and libraries in Eagle (.lbr); Syringe calibration spreadsheet for water and 16% sucrose (.xlsx); Build instructions (.pdf); Bill of materials (.xlsx); Closeup images of the device (.png). Download Extended Data 1, ZIP file. [file sup_enu-eN-OTM-0240-19-s02.zip › Extended Data/finalpinout.png]

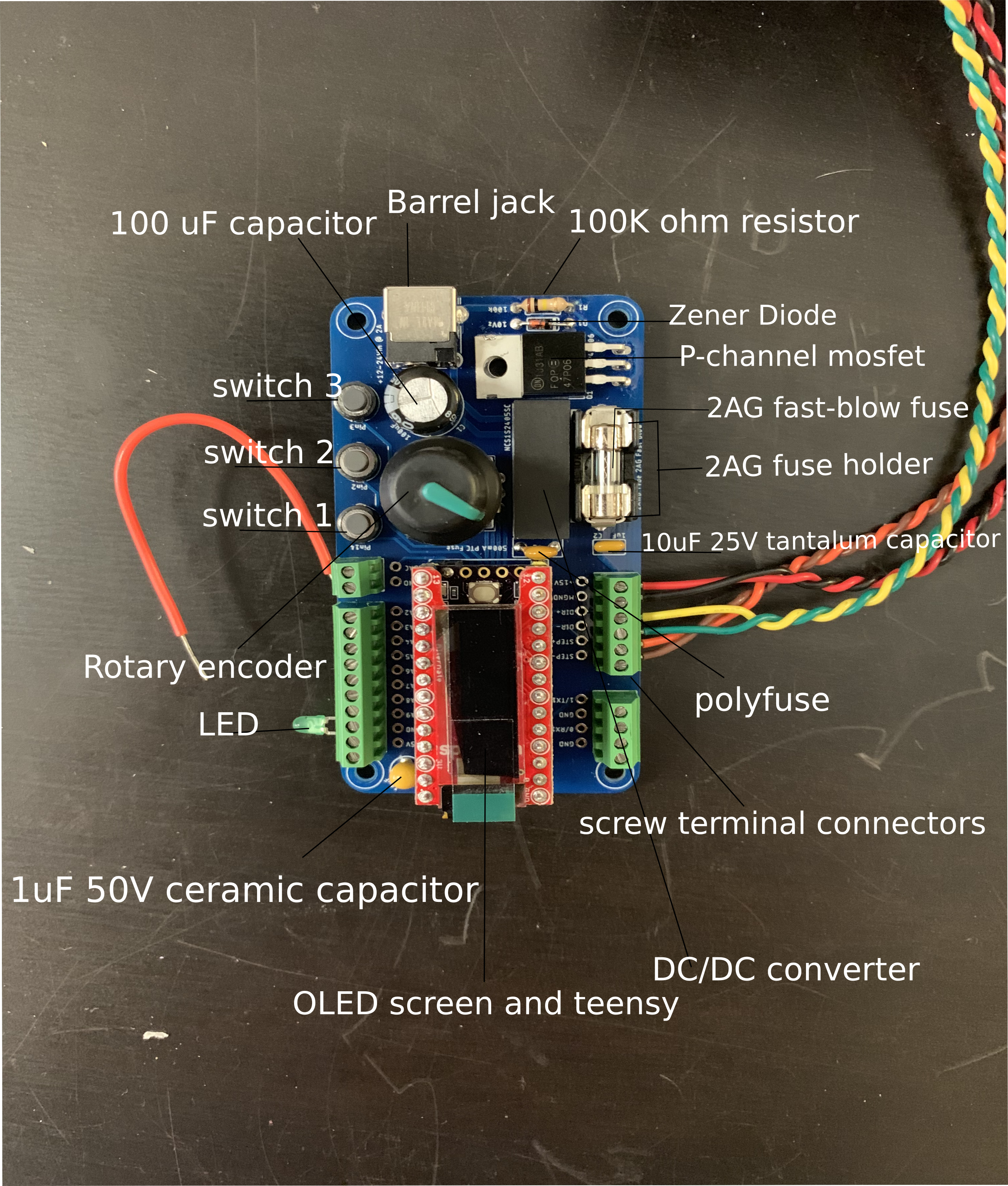

Supplement: Extended Data 1 — Teensy Code Firmware (.ino); PCB design files (.brd, .sch, .pdf) and libraries in Eagle (.lbr); Syringe calibration spreadsheet for water and 16% sucrose (.xlsx); Build instructions (.pdf); Bill of materials (.xlsx); Closeup images of the device (.png). Download Extended Data 1, ZIP file. [file sup_enu-eN-OTM-0240-19-s02.zip › Extended Data/final_labeled_board.png]

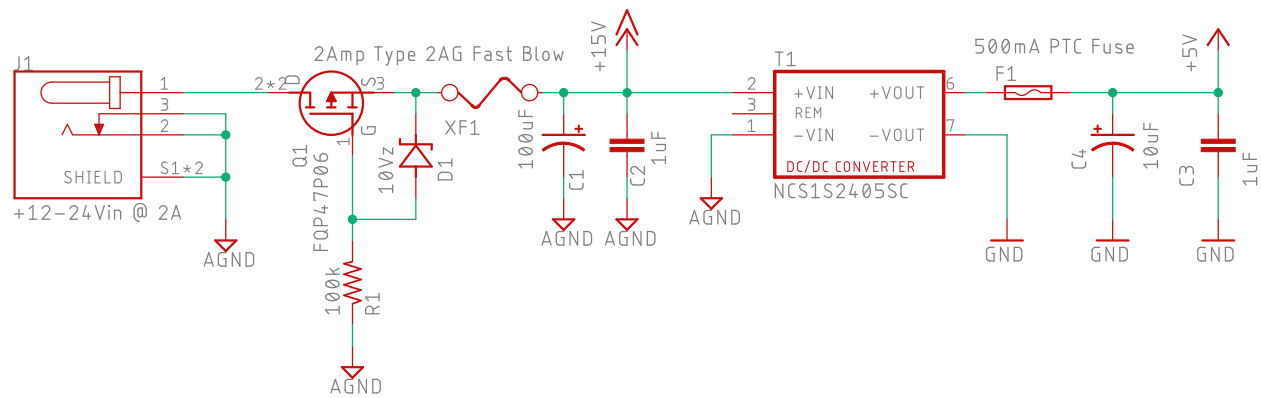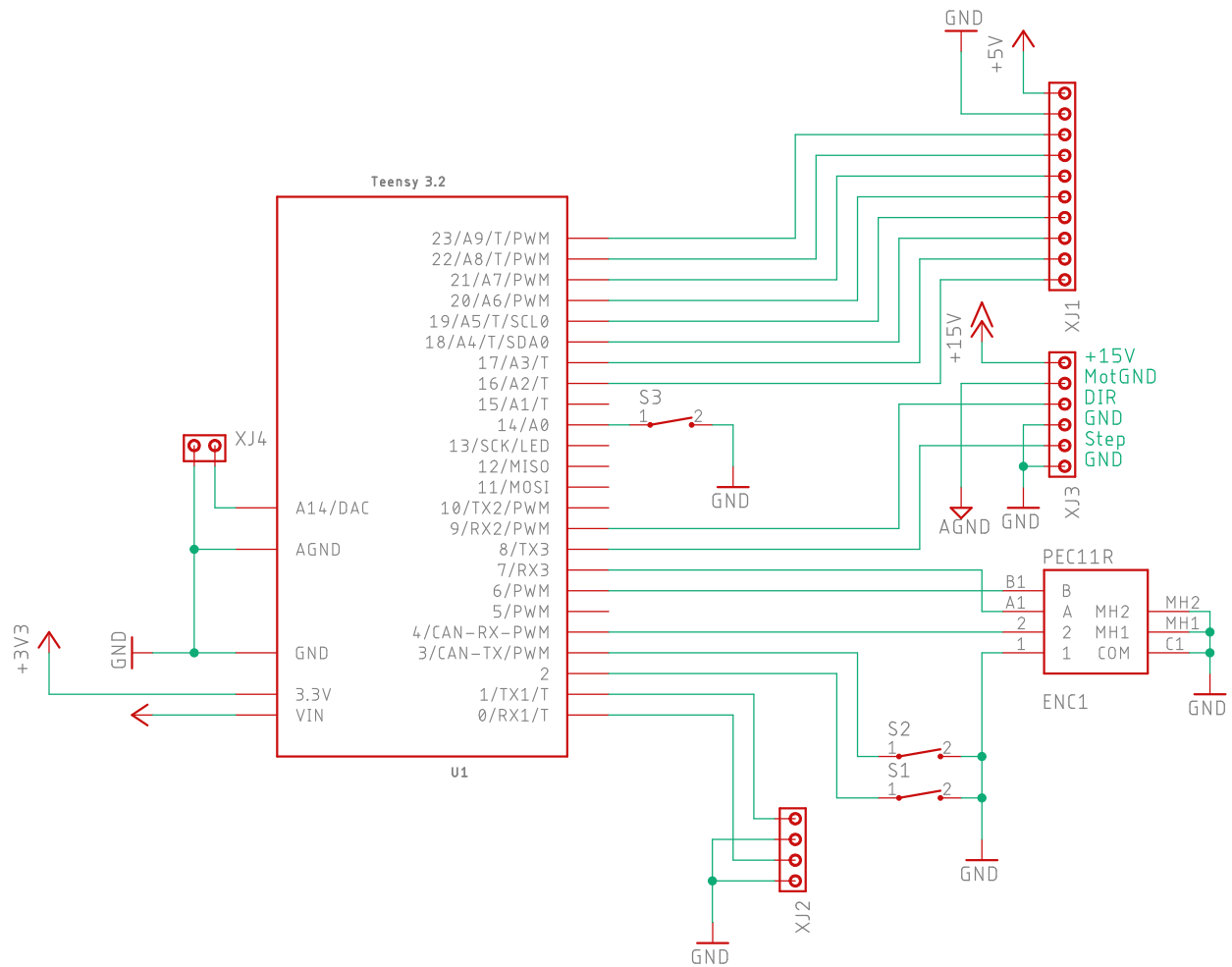

Supplement: Extended Data 1 — Teensy Code Firmware (.ino); PCB design files (.brd, .sch, .pdf) and libraries in Eagle (.lbr); Syringe calibration spreadsheet for water and 16% sucrose (.xlsx); Build instructions (.pdf); Bill of materials (.xlsx); Closeup images of the device (.png). Download Extended Data 1, ZIP file. [file sup_enu-eN-OTM-0240-19-s02.zip › Extended Data/Hardware/OpenSourceSyringePumpSchematic.pdf]

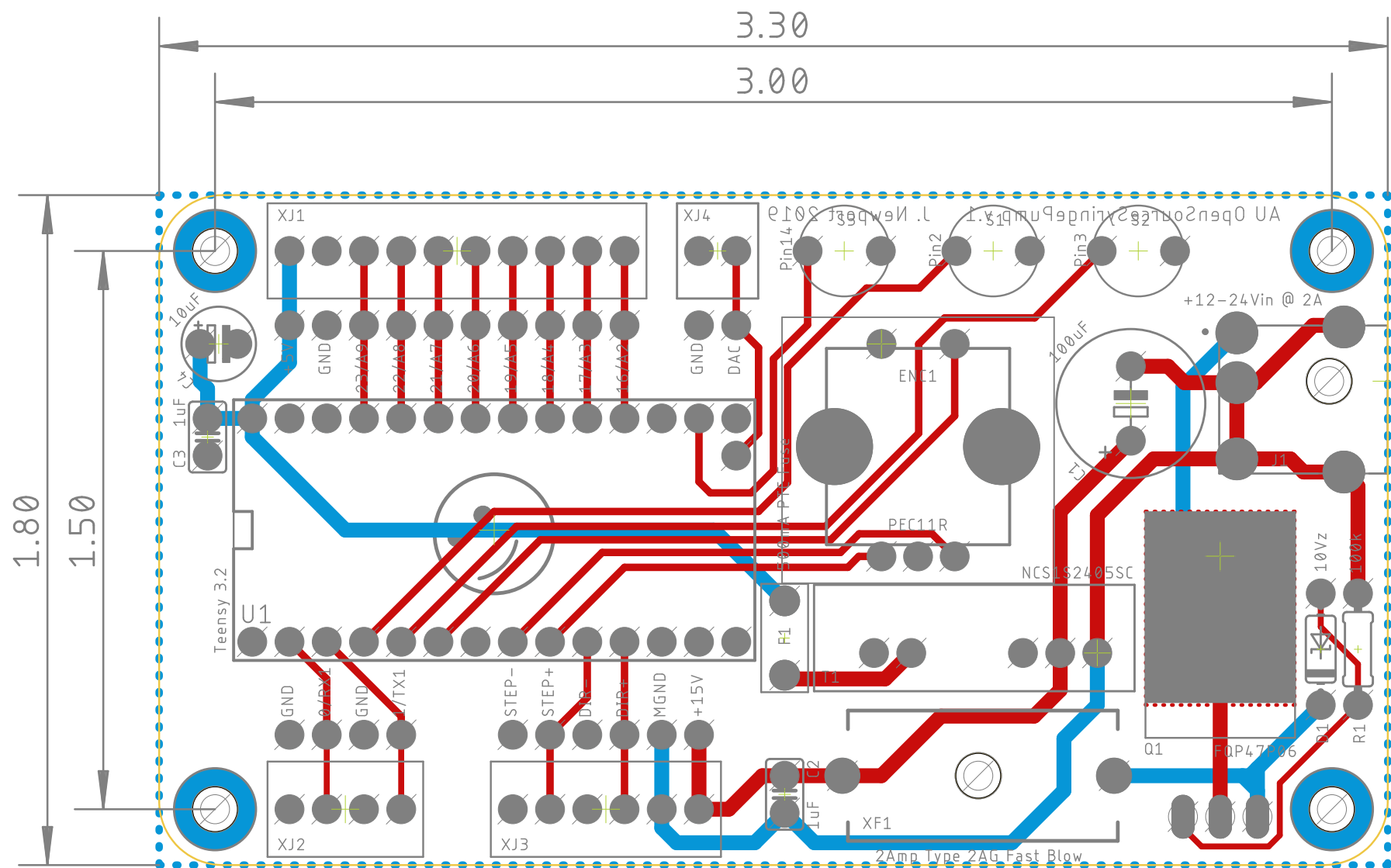

Supplement: Extended Data 1 — Teensy Code Firmware (.ino); PCB design files (.brd, .sch, .pdf) and libraries in Eagle (.lbr); Syringe calibration spreadsheet for water and 16% sucrose (.xlsx); Build instructions (.pdf); Bill of materials (.xlsx); Closeup images of the device (.png). Download Extended Data 1, ZIP file. [file sup_enu-eN-OTM-0240-19-s02.zip › Extended Data/Hardware/OpenSourceSyringePumpPCB.pdf]
